# Diagnosis and outcomes of fever of unknown origin cases with an erythrocyte sedimentation rate of 100 mm/h or more: An International ID-IRI (Infectious Diseases – International Research Initiative) Observational Retrospective Cohort Study

**DOI:** 10.1097/MD.0000000000043341

**Published:** 2025-07-18

**Authors:** Umran Elbahr, Hakan Erdem, Wissal Ben Yahia, Magdalena Petrova Baymakova, Amel Letaief, Kostadin Poposki, Svjetlana Grgić, Gamze Sanlidag, Ionela-Larisa Miftode, Andrea Marino, Egidia Gabriela Miftode, Fatma Amer, Serkan Oncu, Imran Hasanoglu, Ahmed Ashraf Wegdan, Fatma Eser, Hatice Rahmet Guner, Ayse Kaya Kalem, Federica Cosentino, Entela Kolovani, Alper Tahmaz, Meliha Cagla Sonmezer, Jurica Arapovic, Mehmet Resat Ceylan, Bircan Kayaaslan, Atousa Hakamifard, Taylan Önder, Gulden Eser-Karlidag, Hamed Azhdari Tehrani, Syam Kumar Addepalli, Hema Prakash Kumari, Meela Ranjith Kumar, Suresh Babu Sayana, Oguz Resat Sipahi

**Affiliations:** a Infectious Diseases Department, Bahrain Oncology Center, King Hamad University Hospital, Muharraq, Bahrain; b Department of Infectious Diseases and Clinical Microbiology, Turkish Health Sciences University, Gulhane School of Medicine, Ankara, Türkiye; c Internal Medicine Department, Farhat Hached University Hospital, Ibn El Jazzar Medical School, University of Sousse, Sousse, Tunisia; d Department of Infectious Diseases, Military Medical Academy, Sofia, Bulgaria; e Infectious Diseases Department, Farhat Hached University Hospital, Ibn El Jazzar Medical School, University of Sousse, Sousse, Tunisia; f Faculty of Medicine, University Clinic for Infectious Diseases and Febrile Conditions, Skopje, Republic of North Macedonia; g Infectious Diseases Department, University Clinical Hospital Mostar, Mostar, Bosnia and Herzegovina; h Department of Infectious Diseases and Clinical Microbiology, Ege University Faculty of Medicine, Bornova, Izmir, Türkiye; i St. Parascheva Clinical Hospital of Infectious Diseases, Iasi, Romania; j Department of Clinical and Experimental Medicine, Unit of Infectious Diseases, ARNAS Garibaldi Hospital, University of Catania, Catania, Italy; k Department of Medical Microbiology and Immunology, Zagazig University, Faculty of Medicine, Zagazig, Egypt; l Department of Infectious Diseases and Clinical Microbiology, Adnan Menderes University, School of Medicine, Aydin, Türkiye; m Department of Infectious Diseases and Clinical Microbiology, Ankara City Hospital, Ankara, Türkiye; n Department of Medical Microbiology and Immunology, Fayoum University, Faculty of Medicine, Fayoum, Egypt; o University Hospital Center “Mother Theresa,” Infectious Disease Clinic, Tirana, Albania; p Department of Infectious Diseases and Clinical Microbiology, Antalya Training and Research Hospital, Antalya, Türkiye; q Department of Infectious Diseases and Clinical Microbiology, Hacettepe School of Medicine, Hacettepe University, Ankara, Türkiye; r Department of Infectious Diseases and Clinical Microbiology, Harran University, School of Medicine, Sanliurfa, Türkiye; s Infectious Diseases and Tropical Medicine Research Center, Isfahan University of Medical Sciences, Isfahan, Iran; t Department of Infectious Diseases and Clinical Microbiology, Onsekiz Mart University, School of Medicine, Canakkale, Türkiye; u Department of Infectious Diseases and Clinical Microbiology, University of Health Sciences, Elazig Fethi Sekin City Hospital, Elazig Türkiye; v Department of Hematology and Medical Oncology, Shahid Beheshti University of Medical Sciences, Tehran, Iran; w Department of Microbiology, GITAM Institute of Medical Sciences and Research, Visakhapatnam, Andhra Pradesh, India; x Department of Pharmacology, Government Medical College & Government General Hospital, Suryapet, Telangana, India; y Department of Pharmacology, Government Medical College and General Hospital, Bhadradri Kothagudem, Telangana, India.

**Keywords:** erythrocyte sedimentation rate, extremely high ESR, PUO, pyrexia of unknown origin

## Abstract

Herein, we aimed to analyze the final diagnosis in a well-defined cohort of fever of unknown origin (FUO) cases whose erythrocyte sedimentation rate (ESR) was 100 mm/h or more during the admission. The subgroup of the FUO patients with an ESR of 100 mm/h or more during the FUO evaluation, was extracted from the study database of a previously published multicenter study (European Journal of Clinical Microbiology & Infectious Diseases. April 15, 2023;42 (4):387–98). Data for 139 patients (17.6%, 139/788 of the original cohort) who fulfilled the study inclusion criteria, were obtained from 29 centers from 11 countries. Infections, neoplasms, and noninfectious inflammatory diseases were found to be the reason of fever in [n = 74 (53.2%)], [n = 14 (10%)], and [n = 13 (9.3%)] of 139 patients, respectively. Regardless of the diseases subgroup top 6 diseases that were determined to be the reasons of FUO were tuberculosis [n = 15 (10.8%)], HIV/AIDS [n = 13 (9.3%)], urinary tract infection [n = 9 (6.5%)], infective endocarditis [n = 9 (6.5%)], lymphoma [n = 9 (6.5%)], and abscess [n = 9 (6.5%)]. The most common infectious diseases were tuberculosis (15/74, 20.2%), HIV/AIDS (13/74, 17.5%), and infective endocarditis (9/74, 12.1%), along with urinary tract infection [n = 9 (6.5%)] and abscess [n = 9 (6.5%)]. The most common noninfectious inflammatory diseases were adult onset Still disease (3/13, 23%) and giant cell arteritis, also known as temporal arteritis (3/13, 23%), and followed by polyarteritis nodosa (2/13, 15.3%). The most common neoplasm was lymphoma (9/14, 64.2%), followed by lung cancer (2/14, 14.2%). Reason of fever could not be defined in (29/139, 20.8%) patients. The invasive procedures were performed in (64/139, 46%) patients. Gender, age > 50 or not, and income level (high–middle–low) of the participating country were not associated with a significant difference in the final diagnosis category of the FUO case (*P* > .05). To the best of our knowledge, this is the first study evaluating the FUO in the subgroup of cases with extreme ESR elevation and infectious diseases comprised the most cause of the FUO in this particular subgroup.

## 1. Introduction

The term fever of unknown origin (FUO) has been existing in the literature since 1930 with several earlier proposed definitions.^[1]^ However, Petersdorf and Beeson were the first to provide a formal definition of FUO based on a prospective study of 100 cases in 1961.^[2]^ They established the definition of FUO as a fever exceeding 38.3 °C lasting for a minimum of 3 weeks, where the cause remains undiagnosed after a week’s inpatient examination. In 1991, Durack and Street updated the definition of FUO, recommending a shorter diagnostic period of just 3 days, reflecting the advancements in hospital diagnostic capacities.^[3]^ FUO is a diagnostic challenge for physicians because it can stem from more than 100 different diseases, including infections, neoplasms, noninfectious inflammatory diseases (NIID), and miscellaneous, all of which display significant variability.^[4–6]^

The erythrocyte sedimentation rate (ESR) measures the speed how red blood cells (RBC) settle in a given period in unclotted venous blood.^[7]^ The ESR can be raised by a variety of factors such as acute tissue injury, different infections, rheumatologic diseases, neoplasms like lymphoma and lung cancer, which are also potential causes of FUO.^[4,5,7–9]^ Additionally, normal physiological processes like pregnancy may elevate the ESR.^[8]^

ESR has been traditionally utilized by clinicians to gauge the acute and chronic inflammatory phase response, albeit its sensitivity and specificity are low.^[7]^ An ESR that exceeds 100 mm/h is a significant research interest as it usually suggests the existence of a serious underlying condition.^[7–10]^ Despite numerous general and rheumatology-focused studies examining cases with an ESR over 100 mm/h, there is scant data regarding FUO cases with such a high ESR.

Herein, we aimed to analyze the final diagnosis in a well-defined cohort of FUO cases whose ESR was 100 mm/h or more during the admission.

## 2. Materials and methods

### 2.1. Participants and data collection

This research was carried out within the Infectious Diseases – International Research Initiative (ID-IRI) international clinical research platform (available on: https://infectdisiri.com/). This platform has a global membership of clinical researchers who voluntarily participate in ID-IRI.

**Figure 1.**
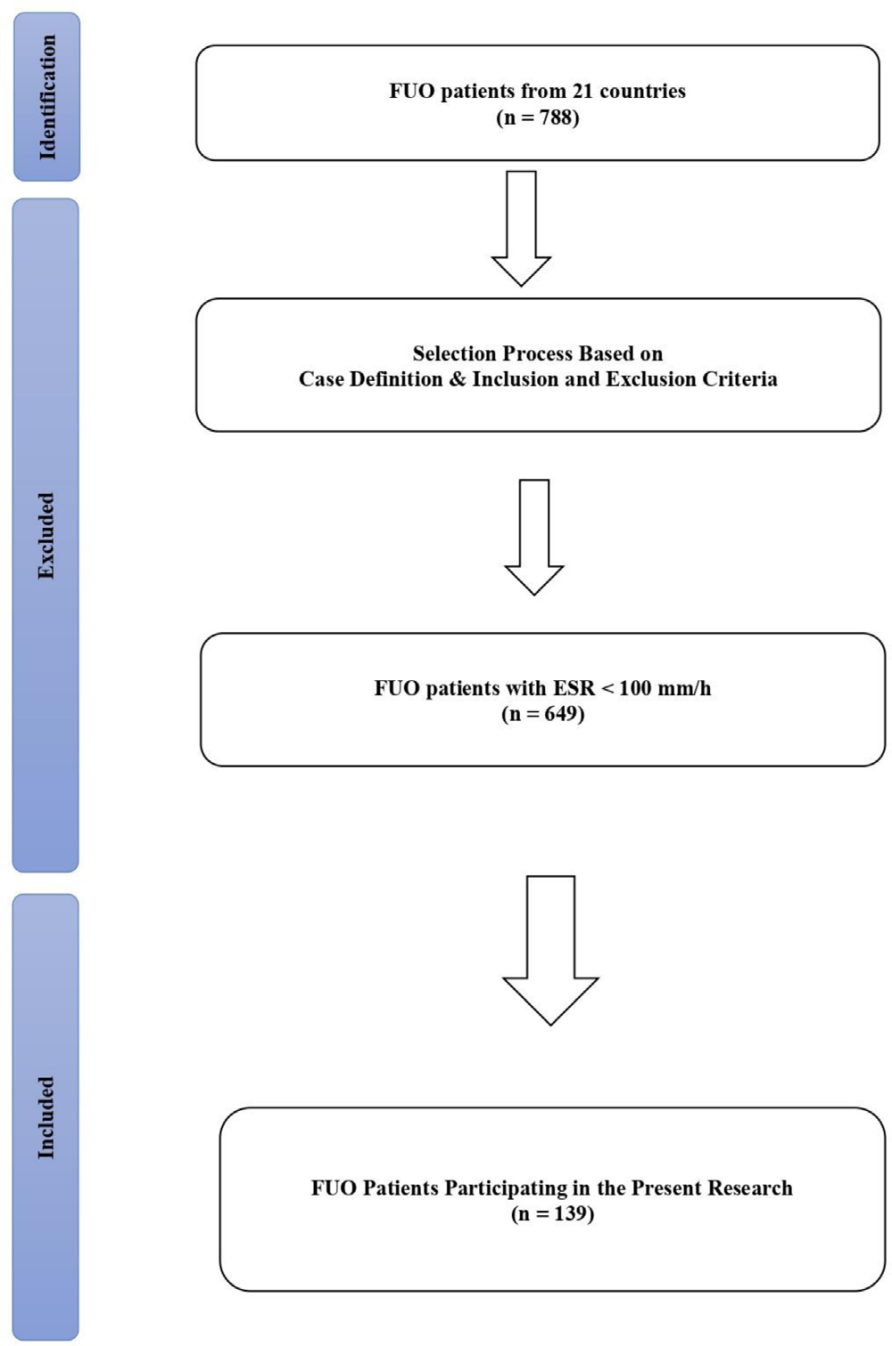
Flow chart showing the selection process of research participants.

The presented international multicenter retrospective cohort study was conducted in line with the Strengthening the reporting of observational studies in epidemiology (STROBE) criteria.^[11]^ We performed a new analysis of an existing large international FUO database which contained 788 cases^[4]^ (Fig. 1). The study sample of this analysis comprised all patients that were admitted due to classical FUO with an ESR of 100 mm/h or more during the FUO evaluation.

Patient data, including demographic details, the duration of the initial FUO evaluation, particulars regarding any laboratory tests or interventions conducted on the patient were entered and stored on a personal password-protected drive to ensure confidentiality.

### 2.2. Case definition

FUO was defined as a febrile condition lasting for more than 3 weeks, with recurrent fevers exceeding 38.3 °C, and the absence of a definitive diagnosis after 3 inpatient days or 3 outpatient visits to a physician as per the study conducted by Durack and Street.^[3]^

### 2.3. Inclusion and exclusion criteria

The inclusion criteria for the present survey were:

- Adults aged 18 and above;
- Patients who were either hospitalized or under medical observation between July 1st, 2016, and July 1st, 2021;
- Patients whose primary clinical symptom was fever (more than 38.3 °C or 100.4 F) and patients fulfilled the above mentioned FUO criteria;
- Having an ESR ≥ 100 mm/h.

The exclusion criteria for the current research include:

- Patients with known immunodeficiency (neutropenia; previously known HIV infection; known hypogammaglobulinemia; hematologic or solid organ transplant; biologic therapies (e.g., anti-TNF or monoclonal antibody) or use of 10 mg prednisone or equivalent for ≥ 2 weeks);
- Pregnant women.

Categorization of the participating countries as per their income was performed as per described in the previous study.^[4]^

### 2.4. Ethical considerations

The research was conducted in accordance with the ethical principles of the Declaration of Helsinki (adopted in June 1964, last revision October 2013). This study granted ethical approval from the Local Ethics Committee of Istanbul Medeniyet University, Faculty of Medicine, Istanbul, Türkiye (August 4, 2021, reference number: 0411), who confirmed that the research was in full accordance with all ethical principles and practices. As this study was a retrospective cohort study, it was not possible to secure informed consent from the participants in the study sample.

### 2.5. Statistical analysis

Data analysis was performed using the Excel 2016 (Microsoft, Redmond) software platform. The data were entered and arranged in MS Excel. Categorical variables were summarized as frequencies and percentages. Comparisons between groups were performed by using the Chi-square test. A *P*-value of < .05 was considered statistically significant. All analyses were performed using SPSS Statistics version [25.0] (IBM Corp., Armonk).

## 3. Results

Data for 139 patients (17.6%, 139/788 of the original cohort) who fulfilled the study inclusion criteria, were obtained from 29 centers from 11 countries. Fifty-four (38.8%) of the cases were female. Mean age was 44 ± 18 years (min 18, max 85, median 48, IQR 39.5–60.5). Mean ESR was 122 ± 56.5 mm/h (min 100, max 763, median 116, IQR 106–127.5).

### 3.1. Distribution of the cases by the countries

The data of patients were submitted from 11 countries: Albania (n = 2; 1.4%), Bosnia and Herzegovina (n = 9; 6.4%), Bulgaria (n = 9; 6.4%), Egypt (n = 8; 5.7%), India (n = 34; 24.4%), Iran (n = 2; 1.4%), Italy (n = 8; 5.7%), North Macedonia (n = 7; 5%), Romania (n = 12; 8.6%), Tunisia (n = 19; 13.6%), and Türkiye (n = 29; 20.8%).

### 3.2. Reasons for FUO

Infections, neoplasms, and noninfectious inflammatory conditions were found to be the reason of FUO in [n = 74 (53.2%)], [n = 14 (10%)], and [n = 13 (9.3%) of 139] patients, respectively. Regardless of the diseases subgroup top 6 diseases that were determined to be the reasons of FUO were tuberculosis [n = 15 (10.8%)], HIV/AIDS [n = 13 (9.3%)], urinary tract infection [n = 9 (6.5%)], infective endocarditis [n = 9 (6.5%)], lymphoma [n = 9 (6.5%)], and abscess [n = 9 (6.5%)]. Reason of fever could not be defined in (29/139, 20.8%) patients. The etiology is summarized in Table 1.

**Table 1:** Etiology of the diseases causing extremely high ESR.

Reason for FUO	Number (percentage)
*Infections*	N = 74
Tuberculosis	15 (20.3%)
HIV positivity	13 (17.6%)
Infective endocarditis	9 (12.2%)
Abscess	9 (12.2%)
Urinary tract infection	9 (12.2%)
Bronchopulmonary infection	5 (6.8%)
Spondylodiscitis	2 (2.7%)
Brucellosis	2 (2.7%)
Bacteraemia	2 (2.7%)
Meningitis	2 (2.7%)
BK virus infection	1 (1.4%)
Rickettsiosis	1 (1.4%)
Q fever	1 (1.4%)
Acute lymphadenitis	1 (1.4%)
Measles	1 (1.4%)
Empyema	1 (1.4%)
*Neoplasms*	N = 14
Hodgkin lymphoma	5 (35.7%)
Non-Hodgkin lymphoma (B cell)	4 (28.6%)
Lung cancer	2 (14.3%)
Adrenal incidentaloma	1 (7.1%)
AML	1 (7.1%)
Renal cancer	1 (7.1%)
*NIID*	N = 13
Adult onset Still disease	3 (23.1%)
Temporal arteritis (giant cell arteritis)	3 (23.1%)
Polyarteritis nodosa	2 (15.4%)
Large vessel vasculitis	1 (7.7%)
Polymyalgia rheumatica	1 (7.7%)
Polymyositis	1 (7.7%)
Systemic lupus erythematosus	1 (7.7%)
Wegener granulomatosis	1 (7.7%)
*Miscellaneous*	N = 9
Thyroiditis	3 (33.3%)
Ulcerative colitis	2 (22.2%)
Histiocytosis	2 (22.2%)
Macrophage activation syndrome	1 (11.1%)
Hemophagocytic syndrome	1 (11.1%)
*Undiagnosed*	N = 29

AML = acute myeloid leukaemia, ESR = erythrocyte sedimentation rate, FUO = fever of unknown origin, HIV = human immunodeficiency virus, NIID = noninfectious inflammatory diseases.

#### 3.2.1. Infections

The most common infectious disease was tuberculosis (15/74, 20.2%) followed by HIV/AIDS (13/74, 17.5%) and infective endocarditis (9/74, 12.1%) along with urinary tract infection [n = 9 (6.5%)] and abscess [n = 9 (6.5%)]. Overall list of the infectious diseases found to be the reason of FUO are summarized in Table 1.

#### 3.2.2. Neoplasms

The most common neoplasm was lymphoma (9/14, 64.2%), followed by lung cancer (2/14, 14.2%).

#### 3.2.3. NIID

The most common noninfectious inflammatory conditions were adult onset Still disease (3/13, 23%) and giant cell arteritis, also known as temporal arteritis (3/13, 23%), followed by polyarteritis nodosa (2/13, 15.3%). Details of the overall noninfectious inflammatory conditions found to be the reason of FUO are summarized in Table 1.

#### 3.2.4. Invasive procedures

The invasive procedures were performed in (64/139, 46%) patients and biopsy was performed in 34 (53.1%) patients. The most common invasive diagnostic procedures were lymph node biopsy [n = 34 (53.1%)], bronchoscopy [n = 17 (26.6%)], and colonoscopy [n = 14 (21.9%)], respectively. The invasive diagnostic interventions performed during investigations is summarized in Table 2.

**Table 2:** The invasive diagnostic procedures performed during fever of unknown origin (FUO) and extremely high erythrocyte sedimentation rate (ESR) investigations.

Invasive procedure (n = 64)	
Biopsy	34 (53.1%)
Lymph node biopsy	34 (53.1%)
Bone marrow biopsy	9 (14.1%)
Temporal artery biopsy	3 (4.7%)
Skin biopsy	2 (3.1%)
Liver biopsy	1 (1.6%)
Lung biopsy	1 (1.6%)
Pleura biopsy	1 (1.6%)
Laparoscopy	1 (1.6%)
Nasal septum biopsy	1 (1.6%)
Sternal biopsy	1 (1.6%)
Bronchoscopy	17 (26.6%)
Colonoscopy	14 (21.9%)
EGD	12 (18.8%)
Peritoneal fluid aspiration	12 (18.8%)
Lumbar puncture	9 (14.1%)
Thoracentesis	5 (7.8%)
Abscess drainage	2 (3.1%)
Arthroscopy for knee	1 (1.6%)

EGD = esophagogastroduodenoscopy.

### 3.3. Distribution of the final diagnosis of FUO according to gender and age

There was no statistically significant difference in the FUO final diagnosis type according to the gender (Table 3, *P* = .305) and based on age groups (<50 vs ≥50 years) (Table 4, *P* = .743).

**Table 3:** Distribution of the final diagnosis categories according to gender.

	Infectious diseases	Neoplasms	Collagen vascular diseases	Undiagnosed fever of unknown origin	Other diseases	Total
Male	46	11	5	17	6	85
Female	28	3	8	12	3	54
Total	74	14	13	29	9	139

**Table 4:** Distribution of the final diagnosis categories according to age groups.

	Infectious diseases	Neoplasms	Collagen vascular diseases	Undiagnosed fever of unknown origin	Other diseases	Total
≤50 aged	44	6	7	14	5	76
>50 aged	30	8	6	15	4	63
Total	74	14	13	29	9	139

### 3.4. Distribution of the final diagnosis of FUO according to the income category of the participating country

When we analyzed the distribution of the FUO final diagnosis categories versus the income category of the participating country there was no significant difference among the groups (Table 5, *P* = .621).

**Table 5:** Distribution of the final diagnosis categories according to age groups.

	Infectious diseases	Neoplasms	Collagen vascular diseases	Undiagnosed fever of unknown origin	Other diseases	Total
Higher income	4	1	1	2	0	8
Middle income	39	5	3	13	3	63
Lower income	31	8	9	14	6	68
Total	74	14	13	29	9	139

## 4. Discussion

ESR has been used as an inflammatory marker in medicine for a very long time. Extremely high ESR is a subject of interest for infectious diseases, neoplasms, or rheumatological diseases. However, there is very scant data regarding FUO cases with extremely high ESR.^[12]^ In this study we analyzed the FUO cases with ≥ 100 mm/h ESR. The most common etiological reasons leading to extremely high ESR were infections, neoplasms, and NIID, respectively. The most common infection was tuberculosis while the most common neoplasm was Hodgkin lymphoma, and the most common NIID was adult onset Still disease along with temporal arteritis. Approximately one-fifth of the patients remained undiagnosed.

Red blood cells carry negative charges on their surface, causing them to repel each other, known as zeta potential. However, many proteins in the plasma have positive charges, which can neutralize the negative charges on RBCs, leading to the formation of rouleaux.^[13]^ An increase in these plasma proteins, common in inflammatory conditions, results in more rouleaux formations, which settle faster than individual RBCs. This constant settling of rouleaux groupings in the Westergren tube contributes to an increase in the ESR.^[14]^

Assessing levels of inflammatory markers such as C-reactive protein, procalcitonin, and ESR may be beneficial for identifying acute inflammation that may signify particular diseases. The inflammatory marker levels tend to rise in relation to a broad range of conditions, including infections, neoplasms, and NIID.^[15]^

In a retrospective, cross-sectional study conducted by Yousuf et al, 508 patients had ESR ≥ 100 mm/hour were evaluated.^[16]^ The leading diagnosis reported as infections (38.6%), autoimmune diseases (15.9%), and malignancy (15.4%).^[16]^ According to their results, the main infections included osteomyelitis, tuberculosis, and urinary tract infections and the main malignancy was lymphoma. The reason behind the extremely high ESR was not identified in fourteen (2.4%) patients.^[16]^ In Fincher and Page study of 1006 patients, most patients with ESR ≥ 100 mm/h had malignancy (33%), rheumatic diseases (17%), and infection (14%).^[17]^ In another retrospective cohort study conducted by Daniels et al, 4807 patients with extreme ESR values were evaluated.^[10]^ The main diagnosis associated with extreme ESR elevations was infection [n = 1932 (40%)], followed by autoimmune/inflammatory diseases [n = 1839 (38%)] and malignancy [n = 1736 (36%)]. This finding is further supported by statistically significant differences observed between the number of cases across these disease categories (*P*-value < .01). Among the patients had autoimmune diseases with the distribution as follows: rheumatoid arthritis (n = 267) was the most common, polymyalgia rheumatica (n = 133), systemic lupus erythematosus (n = 129), giant cell arteritis (n = 106), and gout (n = 101) were the other common rheumatic diseases.^[10]^ The reasons for this significant increase in ESR in patients with the rheumatic disease could be due to the rheumatic disease itself, a flare-up of the underlying disease, or infections added to the clinical picture of the rheumatic diseases.^[18]^ Hence, the characteristics of these 3 conditions are not well documented in the literature. The results we have unearthed in the course of our research align remarkably well with these findings.

Fever of unknown origin presents a significant diagnostic challenge for clinicians due to its diverse etiology. There are numerous studies in the literature aimed at determining the etiology of FUO. In a systematic review analyzed 857 FUO cases from Türkiye revealed that, the most common etiology was infections (47.0%), followed by collagen vascular diseases (15.9%) and neoplasms (14.7%).^[5]^ Similarly, in a multicentre study evaluating the FUO etiology, infections (51.6%; n = 407), malignancy (11.4%, n = 90), and noninfectious collagen vascular disorders (9.3%, n = 73) were reported as the most common etiology by order.^[4]^ In another case series evaluating the etiology of FUO, infections, connective tissue diseases, and neoplasms were found to be the most common causes, with frequencies of 32.7% (n = 32), 14.3% (n = 14), and 18.3% (n = 18), respectively.^[19]^ Considering these data, it can be stated that the etiology of FUO and extremely elevated ESR share similar disease groups.

Previous studies have reported that the proportion of patients have extremely elevated ESR with unknown etiology after investigations is <10%.^[10,17,19]^ In contrast, the etiology remains undetected in nearly 20% of cases with FUO.^[4,15]^ In our study, the proportion of patients without a definitive diagnosis appears to be more consistent with those observed in FUO cases.

The etiology of FUO may vary depending on economic status, and other contextual factors such as healthcare accessibility and diagnostic capabilities. However, in our study, the distribution of the FUO final diagnostic categories versus income type of the participating country as well as age and gender did not result in any significant difference. Further studies may be needed to validate these observations.

This research has some limitations that need to be mentioned. The most significant limitation is its retrospective nature. All potential inherent biases associated with this type of studies may apply to our research. Although this study benefits from its international scope, the findings may not be fully generalizable to settings that were not included. Specifically, the exclusion of pregnant women and patients with known immunodeficiencies means that our results may not apply to these subgroups. Further research is needed to provide a comprehensive understanding of FUO with extremely elevated ESR in these specific populations. The diagnostic approach of FUO was not standard since the data were collected from various centers. The diagnoses for each individual were determined by their respective physicians at specific points in time. Despite these limitations, this survey has several merits. To the best of our knowledge, this is the first study as well as the first international multicenter study that is evaluating the FUO in the subgroup of cases with extreme ESR and similar to FUO, infections comprised the most cause of the cases in this particular subgroup.

## Acknowledgments

This study has been presented as a poster presentation in 34th European Congress of Clinical Microbiology & Infectious Diseases (ECCMID), 27–30 April 2024, Barcelona, Spain. We are grateful to Hilal Sipahi, MD, PhD for statistical analysis.

## Author contributions

**Conceptualization:** Umran Elbahr, Hakan Erdem, Oguz Resat Sipahi.

**Data curation:** Wissal Ben Yahia, Magdalena Petrova Baymakova, Amel Letaief, Kostadin Poposki, Svjetlana Grgić, Gamze Sanlidag, Ionela-Larisa Miftode, Andrea Marino, Egidia Gabriela Miftode, Fatma Amer, Serkan Oncu, Imran Hasanoglu, Ahmed Ashraf Wegdan, Fatma Eser, Hatice Rahmet Guner, Ayse Kaya Kalem, Federica Cosentino, Entela Kolovani, Alper Tahmaz, Meliha Cagla Sonmezer, Jurica Arapovic, Mehmet Resat Ceylan, Bircan Kayaaslan, Atousa Hakamifard, Taylan Önder, Gulden Eser-Karlidag, Hamed Azhdari Tehrani, Syam Kumar Addepalli, Hema Prakash Kumari, Meela Ranjith Kumar, Suresh Babu Sayana.

**Formal analysis:** Umran Elbahr, Wissal Ben Yahia, Magdalena Petrova Baymakova, Amel Letaief, Kostadin Poposki, Svjetlana Grgić, Gamze Sanlidag, Ionela-Larisa Miftode, Andrea Marino, Egidia Gabriela Miftode, Fatma Amer, Serkan Oncu, Imran Hasanoglu, Ahmed Ashraf Wegdan, Fatma Eser, Hatice Rahmet Guner, Ayse Kaya Kalem, Federica Cosentino, Entela Kolovani, Alper Tahmaz, Meliha Cagla Sonmezer, Jurica Arapovic, Mehmet Resat Ceylan, Bircan Kayaaslan, Atousa Hakamifard, Taylan Önder, Gulden Eser-Karlidag, Hamed Azhdari Tehrani, Syam Kumar Addepalli, Hema Prakash Kumari, Meela Ranjith Kumar, Suresh Babu Sayana, Oguz Resat Sipahi.

**Investigation:** Umran Elbahr, Magdalena Petrova Baymakova, Oguz Resat Sipahi.

**Methodology:** Umran Elbahr, Magdalena Petrova Baymakova, Oguz Resat Sipahi.

**Project administration:** Hakan Erdem, Oguz Resat Sipahi.

**Supervision:** Hakan Erdem, Oguz Resat Sipahi.

**Writing – original draft:** Umran Elbahr.

**Writing – review & editing:** Hakan Erdem, Oguz Resat Sipahi.
